# Novel species of *Pestalotiopsis* fungi on *Dracaena* from Thailand

**DOI:** 10.1080/21501203.2020.1801873

**Published:** 2020-08-18

**Authors:** Napalai Chaiwan, Dhanushka N. Wanasinghe, Ausana mapook, Ruvishika S. Jayawardena, Chada Norphanphoun, Kevin D. Hyde

**Affiliations:** a Center of Excellence in Fungal Research, School of Science, Mae Fah Luang University, Chiang Rai, Thailand; b Key Laboratory for Plant Diversity and Biogeography of East Asia, Kunming Institute of Botany, Chinese Academy of Science, Kunming, People’s Republic of China; c Innovative Institute of Plant Health, Zhongkai University of Agriculture and Engineering, Haizhu District, Guangzhou,People’s Republic of China

**Keywords:** 1 new taxon, multigene, phylogeny, saprobe, taxonomy

## Abstract

A survey of the diversity and distribution of microfungi on *Dracaena* leaf litter in Songkhla Province (Thailand) yielded two collections of pestalotiopsis-like fungi. Analyses of a combined ITS, TEF1-α and TUB2 sequence data matrix were applied to infer the phylogenetic position of these new isolates in *Pestalotiopsis*. The phylogenies indicated that these two isolates were monophyletic and constituted a distinct lineage that perceived a taxonomic novelty in *Pestalotiopsis*. This clade shared a close phylogenetic affinity with *P. adusta, P. krabiensis, P. pandanicola* and *P. papuana*. The comparison of morphological features with the phylogenetically closely related taxa are given and the new species is introduced as *Pestalotiopsis dracaenicola* sp. nov. with comprehensive descriptions and illustrations herein.

## Introduction

*Dracaena* is a monocotyledon belonging to the family *Agavaceae* that are used as ornamentals, herbs or medicinal plants (Pires et al. 2004). *Dracaena* consists of about 550–600 species in 18 genera including various shrubs and trees (Pires et al. 2004; Mabberley 2008). Species of *Dracaena* are widely distributed in the tropics and subtropical regions of the world. In Europe and Canada, most *Dracaena* plants are cultivated as ornamentals (Ilodibia et al. 2015). *Dracaena marginata* an important ornamental plant exported as a popular houseplant, has been shown to reduce the levels of formaldehyde in the air (Jaminson 2012). Robiansyah and Hajar (2017) have shown that there is a decline in the population of *D. ombet* throughout its native ranges due to overgrazing, disease by pathogens, human overexploitation, and climate change. The conservation actions for these species are hindered due to poor information about their natural enemies. The plant associated fungi which can be pathogens/opportunistic pathogens, may directly relevant with quarantine measures when the plant is exported as ornamentals to different regions. In contrast to the detailed studies on other hosts such as grasses, bamboo and palms in Thailand, information is still limited on *Dracaena* based fungi. Some taxa occurring on dead leaves of *Dracaena* are *Colletotrichum gloeosporioides* (*D. sanderiana*) (Stevenson 1975), *Gloeosporium* sp. (*D. reflexa*) (Giatgong 1980), *Ophioceras chiangdaoense* (*D. loureiroi*) (Thongkantha et al. 2009), *Parapallidocercospora thailandica* (*D. loureiroi*) (Hyde et al. 2016) and *Phaeosphaeriopsis dracaenicola* (*Dracaena loureiroi*) (Phookamsak et al. 2014). There have been two *Pestalotiopsis* species reported on *Dracaena fragrans: P. affinis* Y.X. Chen & G. Wei and *P. dracaenea* Yong Wang bis, Yu Song, K. Geng & K.D. Hyde.

We are investigating the microfungi associated with monocotyledons in Thailand which has a high species diversity (Dai et al. 2017; Hyde et al. 2018; Tibpromma et al. 2018). In this paper we introduce a novel species in *Pestalotiopsis* from *Dracaena* based on morphology coupled with multigene phylogeny.

## Materials and methods

### Isolates and morphology

*Dracaena* leaf litter was collected from Songkhla Province in Thailand during May 2018. Collected samples were brought to the laboratory in plastic bags. Specimens were observed with a stereomicroscope (Motic SMZ-171). Mycelia or spore mass from specimens was directly isolated on potato dextrose agar (PDA) plates and incubated at 25–30°C. The culture was transferred to new PDA plates. Cultures were grown for 2–4 weeks and morphological characters, such as colour, colony and texture were recorded. The culture characteristics were photographed with a Canon EOS 600D digital camera fitted to a Nikon ECLIPSE Ni compound microscope. Measurements of morphological structures were taken from the widest and the longest parts of each structure. Whenever possible, more than 20 measurements were made. The lengths and widths were measured using the Tarosoft (R) Image Frame Work programme and images used for figures processed with Adobe Photoshop CS6 Extended v. 10.0 (Adobe Systems, USA).

The specimens were deposited in the Herbarium of Mae Fah Luang University (Herb. MFLU) and Culture Collection of Mae Fah Luang University (MFLUCC), Chiang Rai, Thailand. Facesoffungi and Index Fungorum numbers were submitted (Jayasiri et al. 2015; Index Fungorum 2020). New taxa were justified based on guidelines outlined by Jeewon and Hyde (2016).

### DNA extraction, PCR amplification and sequencing

Fungal isolates were grown on PDA media at 25–30°C for 4 weeks. Mycelium was scraped and transferred into 1.5 ml micro centrifuge tubes for genomic DNA extractions. The E.Z.N.A. Forensic DNA Kit (OMEGA® biotek) was used to extract DNA from fungal mycelium. Three loci were amplified, beta-tubulin (TUB2) with primers Bt2a/Bt2b (Glass and Donaldson 1995); internal transcribed spacer region of ribosomal DNA (ITS: ITS5/ITS4) (White et al. 1990) and the translation elongation factor 1-alpha gene (TEF1-α: EF1-728 F/EF1-986 R) (Rehner and Buckley 2005).

The amplification reactions were performed in 25 μl volumes contained of 8.5 μl of sterilised H_2_O, 12.5 μl of Easy Taq PCR Super Mix [mixture of Easy Taq TM DNA Polymerase, dNTPs, and optimised buffer (Beijing Trans Gen Biotech Co., Chaoyang District, Beijing, PR China), 1 μl of each forward and reverse primers (10 pM) and 2 μl of DNA template (1.2 μg/ml)]. The PCR thermal cycle program for ITS and TEF1-α gene amplification was provided as initially 94°C for 3 mins, followed by 35 cycles of denaturation at 94°C for 30 secs, annealing at 55°C for 50 secs, elongation at 72°C for 90 secs, and final extension at 72°C for 10 mins. The PCR thermal cycle program for TUB2 gene amplification was provided as initially 94°C for 3 mins, followed by 35 cycles of denaturation at 95°C for 30 secs, annealing at 53°C for 30 secs, elongation at 72°C for 45 secs, and a final extension at 72°C for 90 secs. The PCR products were sent for sequencing at Sangon Biotech, Shanghai, China.

### Sequence alignment and phylogenetic analyses

**Figure 1.**
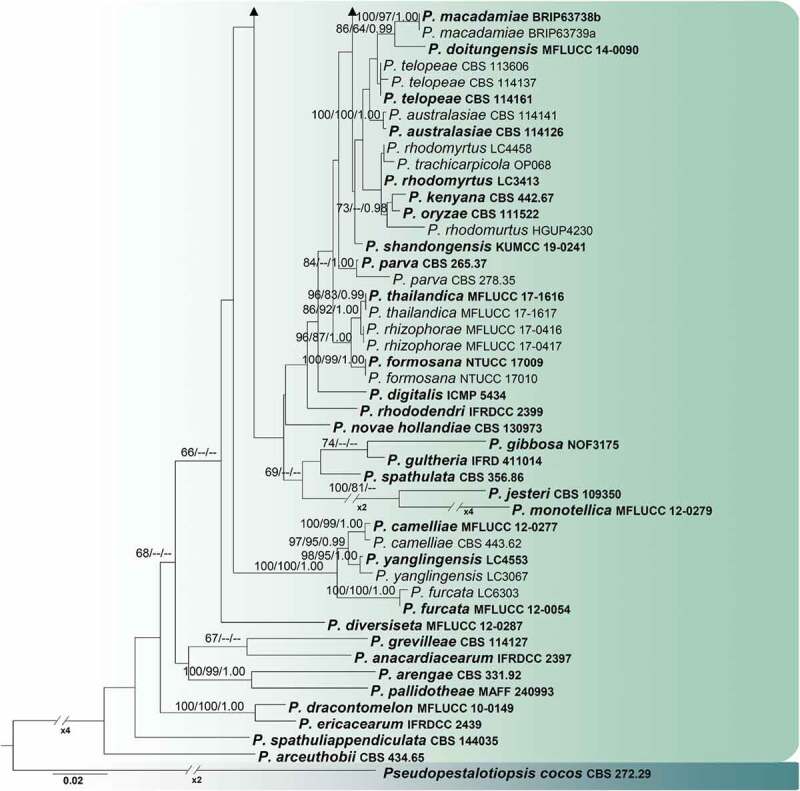
RAxML tree based on analyses of a combined dataset of partial ITS, TEF1-α and TUB2 sequences. Bootstrap support values for ML and MP equal to or greater than 60%, Bayesian posterior probabilities (BYPP) equal to or greater than 0.95 are shown as MP/ML/BI above the nodes. The new isolates are in blue and type species are given in bold. The scale bar represents the expected number of nucleotide substitutions per site

Separate ITS, TEF1-α and TUB2 DNA sequences were subjected to BLAST search engine tool of NCBI for verification and selection of taxa for subsequent phylogenetic analyses. Taxa used in the analyses were obtained from sequence data of *Pestalotiopsis* and related taxa (Table 1) were downloaded from GenBank. Sequence alignments were performed in MAFFT v. 7.220 (mafft.cbrc.jp/alignment/server, Katoh et al. 2017) for each gene locus. Phylogenetic analyses were conducted on a combined dataset of ITS, TEF1-α and TUB2 sequence data. The sequence datasets were combined using BioEdit v.7.2.3 (Hall 1999). Phylogenetic analyses of both individual and combined aligned data were performed under maximum likelihood (ML), maximum parsimony (MP) and Bayesian inference analyses (BI) criteria. Parsimony analysis was carried with the heuristic search option in PAUP (Phylogenetic Analysis Using Parsimony) v. 4.0b10 with the following parameter settings: characters unordered with equal weight, random taxon addition, branch swapping with tree bisection-reconnection (TBR) algorithm, branches collapsing if the maximum branch length was zero. Alignment gaps were treated as missing characters in the analysis of the combined data set, where they occurred in relatively conserved regions. Trees were inferred using the heuristic search option with 1000 random sequence additions, with maxtrees set at 1000. Descriptive tree statistics for parsimony; Tree Length (TL), Consistency Index (CI), Retention Index (RI), Relative Consistency Index (RC) and Homoplasy Index (HI) were calculated for trees generated under different optimality criteria. The Kishino-Hasegawa tests (Kishino and Hasegawa 1989) were performed in order to determine whether trees were significantly different. Maximum parsimony bootstrap values (MP) equal or greater than 60% are given above each node (Figure 1).

**Table 1.** Taxa used in the phylogenetic analyses and their corresponding GenBank numbers. The newly generated sequences are indicated in bold

Species	Culture accession No	GenBank accession			Reference
		ITS	TUB2	TEF1-α	
*Pestalotiopsis adusta*	MFLUCC 10–0146	JX399007	JX399038	JX399071	Maharachchikumbura et al. (2012)
*P. adusta*	ICMP 6088*	AF409957	JX399037	JX399070	Maharachchikumbura et al. (2012)
*P. aggestorum*	LC6301*	KX895015	KX895348	KX895234	Liu et al. (2017)
*P. aggestorum*	LC8186	KY464140	KY464160	KY464150	Liu et al. (2017)
*P. anacardiacearum*	IFRDCC 2397*	KC247154	KC247155	KC247156	Maharachchikumbura et al. (2013)
*P. arceuthobii*	CBS 434.65*	NR147561	KM199427	KM199516	Maharachchikumbura et al. (2014)
*P. arengae*	CBS 331.92*	NR147560	KM199426	KM199515	Maharachchikumbura et al. (2014)
*P. australasiae*	CBS 114,126*	NR147546	KM199409	KM199499	Maharachchikumbura et al. (2014)
*P. australasiae*	CBS 114,141	KM199298	KM199410	KM199501	Maharachchikumbura et al. (2014)
*P. australis*	CBS 111,503	KM199331	KM199382	KM199557	Maharachchikumbura et al. (2014)
*P. australis*	CBS 114,193	KM199332	KM199383	KM199475	Maharachchikumbura et al. (2014)
*P. biciliata*	CBS 124,463*	KM199308	KM199399	KM199505	Maharachchikumbura et al. (2014)
*P. biciliata*	CBS 236.38	KM199309	KM199401	KM199506	Maharachchikumbura et al. (2014)
*P. biciliata*	CBS 790.68	KM199305	KM199400	KM199507	Maharachchikumbura et al. (2014)
*P. brachiata*	LC2988*	KX894933	KX895265	KX895150	Liu et al. (2017)
*P. brachiata*	LC8188	KY464142	KY464162	KY464152	Liu et al. (2017)
*P. brassicae*	CBS 170.26*	KM199379	–	KM199558	Maharachchikumbura et al. (2014)
*P. camelliae*	CBS 443.62	KM199336	KM199424	KM199512	Maharachchikumbura et al. (2014)
*P. camelliae*	MFLUCC 12–0277*	NR120188	JX399041	JX399074	Zhang et al. (2012)
*P. chamaeropis*	CBS 113,607	KM199325	KM199390	KM199472	Maharachchikumbura et al. (2014)
*P. chamaeropis*	CBS 186.71*	KM199326	KM199391	KM199473	Maharachchikumbura et al. (2014)
*P. clavata*	MFLUCC 12–0268*	JX398990	JX399025	JX399056	Maharachchikumbura et al. (2012)
*P. colombiensis*	CBS 118,553*	NR147551	KM199421	KM199488	Maharachchikumbura et al. (2014)
*P. digitalis*	ICMP 5434*	KP781879	KP781883	–	Liu et al. (2015)
*P. diploclisiae*	CBS 115,585	KM199315	KM199417	KM199483	Maharachchikumbura et al. (2014)
*P. diploclisiae*	CBS 115,587*	KM199320	KM199419	KM199486	Maharachchikumbura et al. (2014)
*P. diploclisiae*	CBS 115,449	KM199314	KM199416	KM199485	Maharachchikumbura et al. (2014)
*P. disseminata*	CBS 118,552	MH553986	MH554652	MH554410	Liu et al. (2019)
*P. disseminata*	CBS 143,904	MH554152	MH554825	MH554587	Liu et al. (2019)
*P. disseminata*	CPC 29,351	MH554166	MH554839	MH554601	Liu et al. (2019)
*P. distincta*	LC3232	KX894961	KX895293	KX895178	Liu et al. (2017)
*P. distincta*	LC8184	KY464138	KY464158	KY464148	Liu et al. (2017)
*P. diversiseta*	MFLUCC 12–0287*	JX399009	JX399040	JX399073	Maharachchikumbura et al. (2012)
*P. doitungensis*	MFLUCC 14–0090	MK993573	MK975836	MK975831	Ma et al. (2019)
*P. dracaenae*	HGUP4037*	MT596515	MT598645	MT598644	Ariyawansa et al. (2015)
***P. dracaenicola***	MFLUCC 18–0913*	**MN962731**	**MN962732**	**MN962733**	**This study**
***P. dracaenicola***	**MFLUCC 18–0914**	**MN962734**	**MN962735**	**MN962736**	**This study**
*P. dracontomelon*	MFLUCC 10–0149	KP781877	–	KP781880	Liu et al. (2015)
*P. ericacearum*	IFRDCC 2439*	KC537807	KC537821	KC537814	Zhang et al. (2013)
*P. formosana*	NTUCC 17–009*	MH809381	MH809385	MH809389	Ariyawansa et al. (2018)
*P. formosana*	NTUCC 17–010	MH809382	MH809386	MH809390	Ariyawansa et al. (2018)
*P. furcata*	LC6303	KX895016	KX895349	KX895235	Liu et al. (2017)
*P. furcata*	MFLUCC 12–0054*	JQ683724	JQ683708	JQ683740	Maharachchikumbura et al. (2013)
*P gaultheri*	IFRD 411–014*	KC537805	KC537819	KC537812	Maharachchikumbura et al. (2014)
*P. gibbosa*	NOF 3175*	LC311589	LC311590	LC311591	Watanabe et al. (2018)
*P. grevilleae*	CBS 114,127*	KM199300	KM199407	CBS114127	Maharachchikumbura et al. (2014)
*P. hawaiiensis*	CBS 114,491*	NR147559	KM199428	KM199514	Maharachchikumbura et al. (2014)
*P. hispanica*	CBS 115,391	MH553981	MH554640	MH554399	Liu et al. 2019
*P. hollandica*	CBS 265.33*	NR147555	KM199388	KM199481	Maharachchikumbura et al. (2014)
*P. humus*	CBS 336.97*	KM199317	KM199420	KM199484	Maharachchikumbura et al. (2014)
*P. inflexa*	MFLUCC 12–0270*	JX399008	JX399039	JX399072	Maharachchikumbura et al. (2012)
*P. intermedia*	MFLUCC 12–0259*	JX398993	JX399028	JX399059	Maharachchikumbura et al. (2012)
*P. italiana*	MFLUCC12_0657*	KP781878	KP781882	KP781881	Liu et al. (2015)
*P. jesteri*	CBS 109,350*	KM199380	KM199468	KM199554	Maharachchikumbura et al. (2014)
*P. jiangxiensis*	LC4399*	KX895009	KX895341	KX895227	Liu et al. (2017)
*P. jinchanghensis*	LC6636	KX895028	KX895361	KX895247	Liu et al. (2017)
*P. jinchanghensis*	LC8190*	KY464144	KY464164	KY464154	Liu et al. (2017)
*P. kenyana*	CBS 442.67*	KM199302	KM199395	KM199502	Maharachchikumbura et al. (2014)
*P. krabiensis*	MFLUCC 16–0260	MH388360	MH412722	MH388395	Tibpromma et al. (2018)
*P. knightiae*	CBS 114,138	KM199310	KM199408	KM199497	Maharachchikumbura et al. (2014)
*P. knightiae*	CBS 111,963	KM199311	KM199406	KM199495	Maharachchikumbura et al. (2014)
*P. leucadendri*	CBS 121,417	MH553987	MH554654	MH554412	Liu et al. 2019
*P. licualacola*	HGUP 4057*	KC492509	KC481683	KC481684	Ariyawansa et al. (2018)
*P. linearis*	MFLUCC 12–0271	JX398994	JX399027	JX399060	Maharachchikumbura et al. (2012)
*P. lushanensis*	LC4344*	KX895005	KX895337	KX895223	Liu et al. (2017)
*P. lushanensis*	LC8182	KY464136	KY464156	KY464146	Liu et al. (2017)
*P. macadamiae*	BRIP 63739a	KX186678	KX18668	KX186622	Akinsanmi et al. (2017)
*P. macadamiae*	BRIP 63738b*	KX186588	KX186680	KX186620	Akinsanmi et al. (2017)
*P. malayana*	CBS 102,220*	NR147550	KM199411	KM199482	Maharachchikumbura et al. (2014)
*P. monochaeta*	CBS 144.97*	KM199327	KM199386	KM199479	Maharachchikumbura et al. (2014)
*P. monochaeta*	CBS 440.83	KM199329	KM199387	KM199480	Maharachchikumbura et al. (2014)
*P. montellica*	MFLUCC 12–0279*	JX399012	JX399043	JX399076	Maharachchikumbura et al. (2012)
*P. neglecta*	TAP1100	AB482220	LC311599	LC311600	Norphanphoun et al. (2019)
*P. neolitseae*	NTUCC 17–011*	MH809383	MH809387	MH809391	Ariyawansa and Hyde (2018)
*P. neolitseae*	NTUCC17012	MH809384	MH809388	MH809392	Ariyawansa and Hyde (2018)
*P. neolitseae*	KUMCC 19–0243	MN625276	MN626730	MN626741	Harischandra et al. (2020)
*P. novae-hollandiae*	CBS 130,973*	NR147557	KM199425	KM199511	Maharachchikumbura et al. (2014)
*P. oryzae*	CBS 111,522*	KM199294	KM199394	KM199493	Maharachchikumbura et al. (2014)
*P. oryzae*	CBS 353.69	KM199299	KM199398	KM199496	Maharachchikumbura et al. (2014)
*P. pallidotheae*	MAFF 240,993*	NR111022	LC311584	LC311585	Watanabe et al. (2018)
*P. pandanicola*	MFLUCC 16–0255	MH388361	MH412723	MH388396	Tibpromma et al. (2018)
*P. papuana*	CBS 331.96	KM199321	KM199413	KM199491	Maharachchikumbura et al. (2014)
*P. parva*	CBS 265.37*	KM199312	KM199404	KM199508	Maharachchikumbura et al. (2014)
*P. parva*	CBS 278.35	MH855675	KM199405	KM199509	Maharachchikumbura et al. (2014)
*P. photinicola*	GZcc 16–0028*	KY092404	KY047663	KY047662	Chen et al. (2017)
*P. pinicola*	KUMCC 19–0203	MN412637	MN417508	MN417510	Tibpromma et al. (2019)
*P. pinicola*	KUMCC 19–0183	MN412636	MN417507	MN417509	Tibpromma et al. (2019)
*P. portugalica*	CBS 393.48	KM199335	KM199422	KM199510	Maharachchikumbura et al. (2014)
*P. portugalica*	LC2929	KX894921	KX895253	KX895138	Liu et al. (2016)
*P. rhizophorae*	MFLUCC 17–0416*	MK764283	MK764349	MK764327	Norphanphoun et al. (2019)
*P. rhizophorae*	MFLUCC 17–0417	MK764284	MK764350	MK764328	Norphanphoun et al. (2019)
*P. rhododendri*	IFRDCC 2399	KC537804	KC537818	KC537811	Zhang et al. (2013)
*P. rhodomurtus*	HGUP4230	KF412648	KC537818	KF412645	Song et al. (2013)
*P. rhodomyrtus*	LC3413*	KX894981	KX895313	KX895198	Song et al. (2013)
*P. rhodomyrtus*	LC4458	KX895010	KX895342	KX895228	Liu et al. (2017)
*P. rosea*	MFLUCC 12–0258*	JX399005	JX399005	JX399005	Maharachchikumbura et al. (2012)
*P. scoparia*	CBS 176.25*	KM199330	KM199330	KM199330	Maharachchikumbura et al. (2014)
*P. sequoiae*	MFLUCC 13–0399	KX572339	–	–	Hyde et al. (2016)
*P. shandongensis*	KUMCC 19 0241	MN625275	MN626729	MN626740	Maharachchikumbura et al. (2014)
*P. shorea*	MFLUCC 12–0314*	KJ503811	KJ503814	KJ503817	Song et al. (2104)
*P. spathulata*	CBS 356.86	NR147558	KM199423	KM199513	Maharachchikumbura et al. (2014)
*P. spathuliappendiculata*	CBS 144,035	MH554172	MH554845	MH554607	Liu et al. (2019)
*P. telopeae*	CBS 113,606	KM199295	KM199402	KM199498	Maharachchikumbura et al. (2014)
*P. telopeae*	CBS 114,137*	KM199301	KM199469	KM199559	Maharachchikumbura et al. (2014)
*P. telopeae*	CBS 114,161	KM199296	KM199403	KM199500	Maharachchikumbura et al. (2014)
*P. terricola*	CBS 141.69*	MH554004	MH554680	MH554438	Liu et al. (2019)
*P. thailandica*	MFLUCC 17–1616*	MK764285	MK764351	MK764329	Norphanphoun et al. (2019)
*P. thailandica*	MFLUCC 17–1617	MK764286	MK764352	MK764330	Norphanphoun et al. (2019)
*P. trachicarpicola*	OP068*	JQ845947	JQ845945	JQ845946	Zhang et al. (2012)
*P. unicolour*	MFLUCC 12–0275*	JX398998	JX398998	JX398998	Maharachchikumbura et al. (2012)
*P. unicolour*	MFLUCC 12–0276	JX398999	JX399030	JX399063	Maharachchikumbura et al. (2012)
*P. verruculosa*	MFLUCC 12–0274	JX398996	–	JX399061	Maharachchikumbura et al. (2012)
*P. yanglingensis*	LC3067	KX894949	KX895281	KX895166	Liu et al. (2017)
*P. yanglingensis*	LC4553*	KX895012	KX895345	KX895231	Liu et al. (2017)
*Pseudopestalotiopsis cocos*	CBS 272.29*	MH855069	KM199467	KM199553	Maharachchikumbura et al. (2014)

**Note**: The newly generated sequences are indicated in bold. The type species are noted with a *.

For BI analysis, the best nucleotide substitution model for each locus was identified by comparing the Akaike Information Criterion in MrModeltest v.2.3 (Nylander 2009) and PAUP v.4.0b10 (Swofford 2003) to be (GTR+I + G) for the ITS and TEF1-α, (HKY+I) for the TUB2 alignments. BI analysis was conducted with MrBayes v. 3.1.2 (Huelsenbeck and Ronqvist 2001) to evaluate Bayesian posterior probabilities (BYPP) (Rannala and Yang 1996) by Markov Chain Monte Carlo sampling (BMCMC). GTR+I + G was used in the command. Six simultaneous Markov chains were run for 10,000,000 generations and trees were sampled every 200th generation. The distribution of log-likelihood scores was examined to determine stationary phase for each search and to decide if extra runs were required to achieve convergence, using the program Tracer 1.5 (Rambaut and Drummond 2007). First 20% of generated trees were discarded and remaining 80% of trees were used to calculate posterior probabilities of the majority rule consensus tree. BYPP greater than 0.95 are given above each node (Figure 1).

Maximum likelihood trees were generated using the RAxML-HPC2 on XSEDE (8.2.8) (Stamatakis et al. 2008; Stamatakis 2014) in the CIPRES Science Gateway platform (Miller et al. 2010) using GTR+I + G model of evolution. Maximum likelihood bootstrap values (ML) equal or greater than 60% are given above each node (Figure 1). The phylogenetic trees were shown in FigTree v. 1.4 (Rambaut 2012) and edited using Microsoft Office Power Point 2007 and Adobe illustrator CS3 (Adobe Systems Inc., USA). Sequences derived in this study were deposited in GenBank (Table 1). The finalised alignment and tree were deposited in TreeBASE, submission ID: 26152.

## Results and discussion

### Phylogenetic analyses

The combined sequence alignment of *Pestalotiopsis* comprised 115 taxa, including *Pseudopestalotiopsis cocos* (CBS 272.29) as the outgroup taxon. The dataset included 1486 characters (ITS: 1 to 571 bp, TEF1-α: 572 to 1056 bp, TUB2: 1057 to 1486 bp), after the alignment. Tree topologies (generated under ML, MP and Bayesian criteria) from single gene datasets were also compared and the overall tree topology was congruent to those obtained from the combined dataset of ML tree (Figure 1). The RAxML analysis of the combined dataset yielded a best scoring tree (Figure 1) with a final ML optimisation likelihood value of −13,588.11947. The matrix had 667 distinct alignment patterns, with 7.06% of undetermined characters or gaps. Parameters for the GTR + I + G model of the combined ITS, TEF1-α and TUB2 were as follows: Estimated base frequencies; A = 0.246189, C = 0.263688, G = 0.243646, T = 0.246477; substitution rates AC = 1.335541, AG = 3.561498, AT = 1.209470, CG = 1.017519, CT = 5.175761, GT = 1.000000; gamma distribution shape parameter α = 0.763268. The phylogenetic tree obtained in this study showed similar results to previous studies (Tibpromma et al. 2019). The maximum parsimonious dataset consisted of which 924 constants, 395 (42.74%) parsimony-informative and 173 parsimony-uninformative characters. The parsimony analysis of the data matrix resulted in all equally most parsimonious trees with a length of 2171 steps (CI = 0.384, RI = 0.691, RC = 0.265, HI = 0.616) in the first tree. The Bayesian analysis resulted in 50,001 trees after 10,000,000 generations. The first 10,000 trees, representing the burn-in phase of the analyses, were discarded, while the remaining 40,001 trees were used for calculating posterior probabilities in the majority rule consensus tree. Phylogram depicts that our two strains (MFLUCC 18–0913 and MFLUCC 18–0914) constitute an independent and strongly supported subclade (100% ML and MP, 1.00 BYPP) within the genus *Pestalotiopsis*, sharing a close affinity to *P. adusta* (Ellis & Everh.) Steyaert, *P. krabiensis* Tibpromma & K.D. Hyde, *P. pandanicola* Tibpromma & K.D. Hyde and *P. papuana* Maharachch., K.D. Hyde & Crous (Subclade A1, Figure 1).

**Figure:**
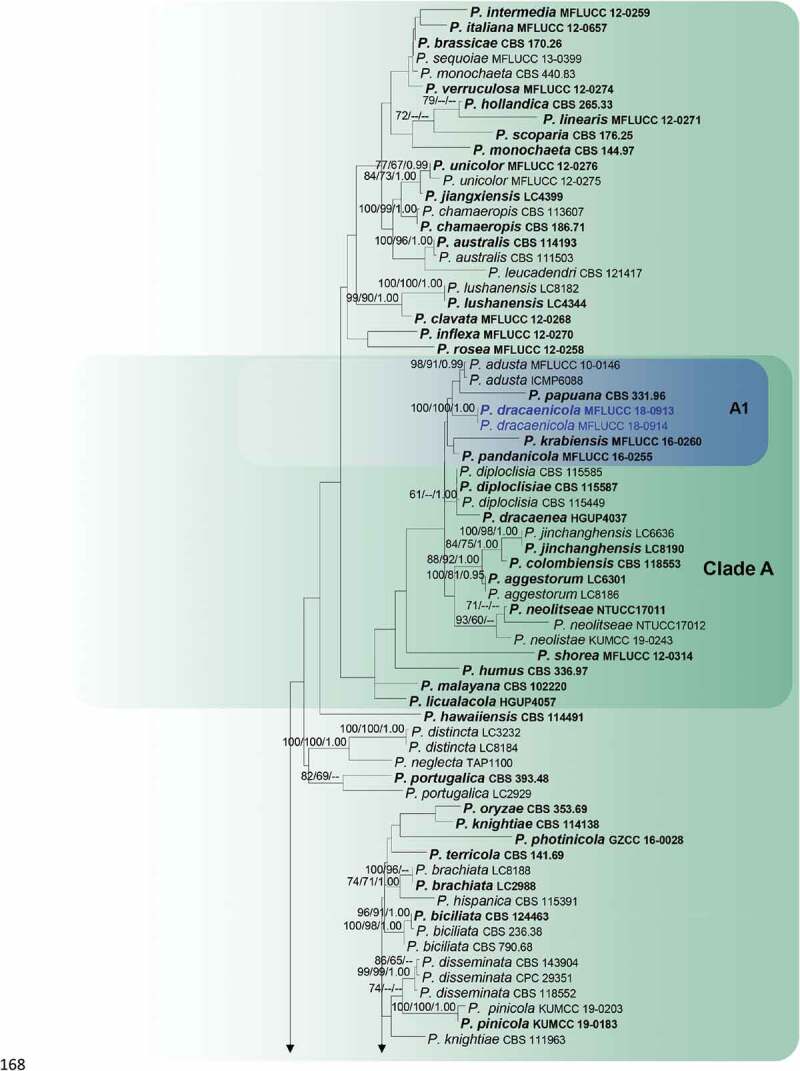

## Taxonomy

***Pestalotiopsis dracaenicola*** Chaiwan & K.D. Hyde, sp. nov.

Index Fungorum number: IF557787; Facesoffungi number: FoF08710Etymology – Name reflects the host genus, *Dracaena*.Holotype: MFLU 19–2905

##### *Saprobic* or *endophytic* on *Dracaena*

**Figure 2.**
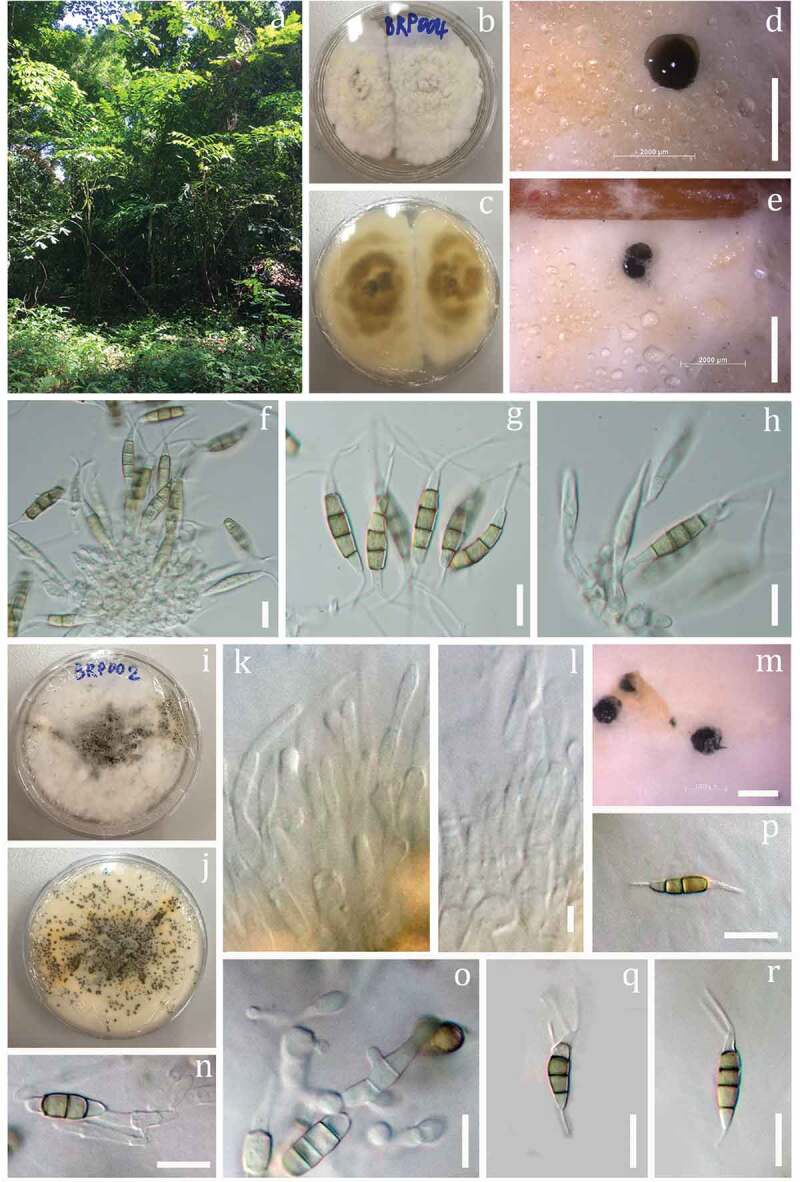
*Pestalotiopsis dracaenicola*. (**b-h** the morphology from MFLUCC 18–0914) (**i-q** the morphology from MFLUCC 18–0913) **a** Habitat. **b, c** Culture on PDA (MFLUCC 18–0914). **d, e**. Colony sporulating on PDA. **f, g, h** Conidiogenous cell with conidia. **i, j** Culture on PDA (MFLUCC 18–0913, **ex-type**). **k, l** Conidiogenous cell. **m** Colony sporulating on PDA. **n, o**. Conidiogenous cell with conidia. **p, q, r** Conidia. Scale bars: d, e = 2000 µm, l = 1000 µm, f-h, k, m-q = 10 μm

**Sexual morph**: Undetermined. **Asexual morph**: *Conidiomata* (on PDA) pycnidial, globose to clavate, solitary, 800–1000 μm (*x̄* = 900 *n* = 20) diam., exuding globose, dark brown to black conidial masses. *Conidiophores* indistinct often reduced to conidiogenous cells. *Conidiogenous cells* discrete, subcylindrical to ampulliform, hyaline. *Conidia* 22–26 × 4–6 μm (*x̄* = 24 × 5 μm, *n* = 30), fusoid, ellipsoid, straight to slightly curved, 4-septate, basal cell conic with a truncate base, hyaline and thin-walled, 2–5 μm long (*x̄* = 3.5 μm, *n* = 30); three median cells doliiform, 13–15 μm long (*x̄* = 14 μm, *n* = 30), wall smooth, concolourous, septa darker than the rest of the cell (second cell from the base pale brown, 4–5 μm long; third cell, 3–5 μm long; fourth cell, 3–4 μm long); apical cell 2–3 (*x̄* = 2.5 μm, *n* = 30) long, hyaline, subcylindrical, thin- and smooth-walled; with 1–3 tubular apical appendages (mainly 2 tubular appendages) 6–11 μm long (*x̄* = 8.5 μm, *n* = 30), arising from the apical crest, unbranched, filiform; basal appendage 3–5 μm long (*x̄* = 4 μm, *n* = 30), single, tubular, unbranched, centric (Figure 2).

##### Culture characteristics

Conidia germinating on PDA within 12 hours reaching 6 cm diameter after 6 days at 25–30°C, circular, floccose to fluffy; white mycelium with aerial on the surface, producing black spore masses.

##### Material examined

THAILAND, Songkhla Province, on dead leaves of *Dracaena* sp. (Asparagaceae), 9 May 2018, Napalai Chaiwan, BRP002 (MFLU 19–2905, **holotype**), ex-type living culture, MFLUCC 18–0913, *ibid*. BRP004 (MFLU 19–2906).

##### Notes

*Pestalotiopsis dracaenicola* has a close phylogenetic affiliation to *P. adusta* (ICMP6088, MFLUCC 16–0255), *P. krabiensis* (MFLUCC 16–0260), *P. pandanicola* (MFLUCC 16–0255) and *P. papuana* (CBS 331.96). *Pestalotiopsis dracaenicola* differs from *P. adusta, P. krabiensis, P. pandanicola* and *P. papuana* in having different sizes of morphological features and the number of apical appendages (Table 2). Meanwhile, *Pestalotiopsis adusta* was reported on leaves of *Prunus cerasus* in USA, from a PVC gasket of a refrigerator door and from *Syzygium* species in Thailand (Maharachchikumbura et al. 2012). *Pestalotiopsis krabiensis* and *P. pandanicola* were found on *Pandanus* sp. in Thailand (Tibpromma et al. 2018). *Pestalotiopsis dracaenea* (HGUP4037) and *Pestalotiopsis affinis* (Hsp2000 II-6600) also found on *Dracaena* (*D. fragrans*) from China (Chen et al. 2002; Ariyawansa et al. 2015).

**Table 2.** Comparison of conidia of *Pestalotiopsis* species related to this study

Species	Conidia Size (μm)	Three median cells of conidia (μm)				Apical appendages		Basal appendage (μm)	References
		Sum of three median cells	second	third	fourth				
						Number	Length (μm)		
*Pestalotiopsis adusta*	16–20 × 5–7	12.4–13.8	4.3–5.3	4–4.7	3.8–4.4	2–3	7–15	–	Maharachchikumbura et al. (2012)
*P. affinis*	17.5–25.2 × 6.3–6.9	13–14	2–4	3–4	3–4	3	13–14	1–3	Chen et al. (2002)
*P. dracaenea*	18–24 × 6.5–8.5	11.5–16	3.5–5.5	4–5.5	4–5.5	2–4	6.5–15.5	unequal	Maharachchikumbura et al. (2012)
*P. dracaenicola*	22–26 × 4–6	13–15	4–5	3–5	3–4	1–3	6–11	3–5	This study
*P. krabiensis*	19–25 **× **4–6	13– 15	3–5	4–5.5	4–5	2–3	11–19	1	Tibpromma et al. (2018)
*P. pandanicola*	13–18 × 2.5–4.5	8–11	2–4	2.5–4	2.5–4	2–3	9.5–26	1	Tibpromma et al. (2018)
*P. papuana*	18–22 × 6–7.5	12–15	3.5–5.5	4.5–5.5	4.5–6	1–2	1.5–7	0.5–2	Maharachchikumbura et al. (2014)

*Pestalotiopsis affinis* (Hsp2000 II-6600) only known from its morphological descriptions and there are no DNA based sequence data to compare the phylogenetic relationship with our new species. *P. dracaenea* (HGUP4037) is not monophyletic with *Pestalotiopsis dracaenicola* (Figure 1).

Comparison of TEF1-α and TUB2 sequences between our fungi and *P. dracaenea* (HGUP4037), showed that they are different 11 bp (2.47%) in 446 TEF1-α nucleotide and 8 bp (1.99%) in 402 TUB2 nucleotide (Table 3). Both *P. dracaenea* (HGUP4037) and *P. affinis* (Hsp2000 II-6600) presence broader conidia than our new species (*P. dracaenicola*: 22–26 × 4–6 μm, *P. dracaenea*: 18–24 × 6.5–8.5 μm and *P. affinis*: 17.5–25.2 × 6.3–6.9 μm), but our species thinner and slander than these two species (Table 2). Our new species also differ from the number of apical appendages, *P. dracaenicola* number of apical appendages 1–3 and length 6–11 μm, while *P. dracaenea* number of apical appendages 2–4 and length 6.5–15.5 μm and *P. affinis* number of apical appendages 3 and length 13–14 μm (Table 2).

**Table 3.** TEF1-α and TUB2 gene character comparisons of *Pestalotiopsis* species used in this study

Taxon/Character	TEF1-α											TUB2							
	17	37	48	61	80	90	165	178	235	379	412	57	232	241	314	368	381	389	396
*P. dracaenicola* (18–0913)	T	-	G	-	T	C	G	C	T	T	A	G	C	C	C	C	T	C	G
*P. dracaenicola* (18–0914)	T	-	G	-	T	C	G	C	T	T	A	G	C	C	C	C	T	C	G
*P. dracaenea* (HGUP4037)	C	T	T	G	C	A	A	G	A	A	G	A	G	T	-	G	-	T	-
